# Risk factors and their impact on carotid intima-media thickness in young and middle-aged ischemic stroke patients and controls: The Norwegian Stroke in the Young Study

**DOI:** 10.1186/1756-0500-7-176

**Published:** 2014-03-26

**Authors:** Annette Fromm, Øystein Ariansen Haaland, Halvor Naess, Lars Thomassen, Ulrike Waje-Andreassen

**Affiliations:** 1 Centre for Neurovascular Diseases, Department of Neurology, Haukeland University Hospital, Bergen, Norway; 2 Department of Clinical Medicine, University of Bergen, Bergen, Norway; 3 Department of Global Public Health and Primary Health Care, University of Bergen, Bergen, Norway; 4 Centre for age-related medicine, Stavanger University Hospital, Stavanger, Norway

**Keywords:** Young stroke, Ischemic stroke, Risk factors, Carotid intima-media thickness, Atherosclerosis, Ultrasound

## Abstract

**Background:**

Vascular morbidity and mortality due to cardiovascular disease (CVD) are high after ischemic stroke at a young age. Data on carotid intima-media thickness (cIMT) as marker of atherosclerosis are scarce for young stroke populations. In this prospective case–control study, we examined cIMT, the burden of vascular risk factors (RF) and their associations among young and middle-aged ischemic stroke patients and controls. We aimed to detect clinical and sub-clinical arterial disease.

**Methods:**

This study was conducted in 150 patients aged 15–60 years and 84 controls free of CVD. We related RF to ultrasonographic B-mode cIMT-measurements obtained from 12 standardized multiangle measurements in the common carotid artery (CCA), carotid bifurcation (BIF) and internal carotid artery (ICA).

**Results:**

RF burden was higher among patients than among controls (p < 0.001). In multivariate analyses of all 234 participants, increased cIMT was associated with age in each carotid segment. Incident stroke was associated with increased ICA-IMT. ICA-IMT increase was associated with a family history of CVD among patients aged 15–44 years, and with RF at mid-age. The overall cIMT difference between patients and controls was 12% for CCA, 17% for BIF and 29% for ICA. Further, increased CCA-IMT was associated with male sex and hypertension. Increased BIF-IMT was associated with dyslipidemia, coronary heart disease and smoking. Increased ICA-IMT was associated with dyslipidemia and stroke.

**Conclusions:**

Ischemic stroke is associated with increased ICA-IMT, related to a family history of CVD among patients aged <45 years, and to increasing RF burden with increasing age. Preventive strategies and aggressive RF treatment are indicated to avoid future cardiovascular events.

**Trial registration:**

NOR-SYS is registered in ClinicalTrials.gov (NCT01597453).

## Background

High rates of recurrent stroke, vascular morbidity and mortality due to cardiovascular disease (CVD) [1-3] clarify the need to detect and treat vascular risk factors and incipient atherosclerosis at early stages. Carotid intima-media thickness (cIMT) is a surrogate marker of atherosclerosis [4,5], and ultrasound screening a valuable tool for cardiovascular risk prediction [6,7]. Nevertheless, cIMT data obtained from young stroke populations are scarce. We aimed to assess the prevalence of clinical and subclinical carotid artery atherosclerosis and the impact of vascular risk factors (RF) among young ischemic stroke patients compared to CVD-free controls in a case–control study.

## Methods

The Norwegian Stroke in the Young Study (NOR-SYS) is a prospective three-generation study with longitudinal follow-up design. NOR-SYS combines medical history and RF ascertainment by standardized questionnaires with clinical, laboratory, neuroradiological, cardiological and complex ultrasonographic data [8]. This analysis contains data from 150 patients and 84 controls included in NOR-SYS between September 2010 and June 2012.

### Approvals, registrations and consents

NOR-SYS is conducted according to the Declaration of Helsinki, approved by the Regional Committee for Medical and Health Research Ethics, Western-Norway (2010/74), and registered in ClinicalTrials.gov (NCT01597453). Written informed consent was obtained from all participants or their legal representatives.

### Subject selection

Patients aged 15–60 years with documented acute ischemic stroke and residency in Hordaland county, Norway, were included. Two patients refused study participation. Seven patients were excluded; three due to incomplete neurosonographic data set, and four non-Caucasian patients. Patients’ partners served as controls due to their function as reference persons for joint offspring in future analyses. Of 123 available partners, 63 (70.8%) females and 21 (61.8%) males participated. Seven controls with prior cardiovascular events were excluded from statistical analysis.

### Risk factors

Medical history of prior stroke, coronary heart disease (CHD: myocardial infarction, angina pectoris) and peripheral artery disease (PAD) was defined if diagnosed before admission or revealed during hospitalization for the qualifying stroke. Family history of CVD (stroke, CHD and/or PAD) was considered positive if reported for parents and/or siblings. Hypertension and diabetes mellitus were defined by diagnosis and/or treatment before hospital admission, or when revealed and treated during hospitalization for the qualifying stroke (blood pressure >140/90 mmHg; HbA1c >6.4%). Dyslipidemia was defined as prior statin use, or when revealed during hospitalization (total cholesterol >5.0 mmol/L and/or low-density lipoprotein (LDL) >3.0 mmol/L and/or high-density lipoprotein (HDL) <1,0 mmol/L and/or triglycerides >2.5 mmol/L). Smoking was categorized as never-smoking or previous/current smoking. Alcohol consumption was categorized as never used/low (0–12 units/week) or high (>12 units/week). Body-mass index (BMI) was dichotomized as normal or >25. RF burden was defined as the number of RFs present (0–10).

### Neurosonology and Duplex/Doppler ultrasound examinations

Extracranial high-resolution sonography of the carotid arteries was performed with Philips iU22 and 9–3 MHz linear array transducer. Two patients were examined at the intensive care unit with a portable Phillips CX50 ultrasound system and 12–3 MHz linear array transducer (both systems Philips Medical Systems, Bothell, WA, USA). Patients and controls were examined by two sonographers (AF, UWA), which both are trained and certified for the NOR-SYS duplex sonography research protocol in collaboration with the Vascular Imaging Centre, University Medical Centre, Utrecht, The Netherlands.

#### Data reliability tests

Reproducibility testing of cIMT measurements within (intra-observer) and between (inter-observer) sonographers of the research group, and between ultrasound equipment (inter-equipment) was performed applying both ultrasound systems. The intra-observer correlation of sonographers was 0.78-0.98 (mean absolute cIMT difference 0.02-0.08 mm). The inter-observer correlation of sonographers was 0.83-0.93 (mean absolute cIMT difference 0.04-0.11 mm). The inter-equipment correlation for iU22/CX50 was 0.94 (mean absolute cIMT difference 0.04 mm). These results correlate with previously published studies [9-12].

#### cIMT-measurements

The methods of the cIMT measurements were previously described [8]. In total 12 far-wall cIMT measurements in the common carotid artery (CCA), the carotid bifurcation (BIF) and the internal carotid artery (ICA) were performed in each participant in the end-diastolic phase of the cardiac cycle, and mean cIMT values were acquired using Philips QLAB® (Philips Medical Systems, Bothell, WA, USA). In case of intra-segmental irregularities or plaques, maximum IMT or plaque thickness were measured additionally. Maximum segmental IMT values were used in statistical analysis. IMT values were defined as normal when <0.8 mm, as suspect for arterial disease when 0.8-0.99 mm [13,14], and as pathological when ≥1.0 mm [15,16]. Plaques were defined as focal IMT measurements >1.5 mm [17].

### Statistical analyses

To allow for comparison to other studies [18-20], and in order to assess the influence of age and sex on IMT, our study population was grouped according to age (younger, 15–44 years; middle-aged, 45–60 years) and sex. Statistical analysis was performed using R version 3.0.0, and data were formatted in STATA version 12.1. A t-test was applied when testing for differences between groups. The unadjusted analysis was conducted applying a univariate linear regression. Because each individual had two measurements per segment (right/left), a random intercept approach was used, utilizing the R-function lmer() from the lme4 package. Dependent variables (cIMT) were skewed towards low values, and a base 10 log transform was applied to meet the assumptions of a linear regression. Hence, the relative change (RC) in cIMT per unit change in the independent variables (typically 0 or 1) could be obtained. Adjusted analyses were carried out accordingly, and multivariate logistic regression was applied. Simulations were used to determine the power to detect differences between controls and patients for RC ranging from 1.00 to 1.50 across each carotid segment. Fisher’s exact test (based on simulations when appropriate) was applied when comparing tables or rows within tables.

## Results

Population demographics are given in Table 1. Of patients, 30.0% were aged 15–44 at study inclusion, and 32.7% were female. Of 84 controls, 25.0% were aged 15–44 at study inclusion, and 75.0% were female.

**Table 1:** Patient and control characteristics

		**All**	**15-44 y**	**45-60 y**	**Females**	**Males**	**Age (p)**	**Sex (p)**
**Patients**	**N**	150(100)*	45(30)*	105(70)*	49(32.7)*	101(67.3)*		
Age (mean)	150	48.5	35.8	54.0	46.3	49.6	<0.001	0.075
Prior stroke	150	13(8.7)	1(2.2)	12(11.4)	6(12.2)	7(6.9)	0.017	0.325
CHD	150	15(10.0)	1(2.2)	14(13.3)	4(8.2)	11(10.9)	0.006	0.589
PAD	150	8(5.3)	1(2.2)	7(6.7)	2(4.1)	6(5.9)	0.181	0.617
Family CVD	150	78(52.0)	9(20.0)	69(65.7)	29(59.2)	49(48.5)	<0.001	0.222
Diabetes	150	16(10.7)	2(4.4)	14(13.3)	7(14.3)	9(8.9)	0.053	0.357
Hypertension	150	101(67.3)	23(51.1)	78(74.3)	30(61.2)	71(70.3)	0.009	0.282
Dyslipidemia	150	114(76.0)	27(60.0)	87(82.9)	36(73.5)	78(77.2)	0.007	0.623
Smoking	150	104(69.3)	23(51.1)	81(77.1)	28(57.1)	76(75.2)	0.003	0.033
BMI > 25	144	99(68.8)	28(62.2)	71(67.6)	30(61.2)	69(68.3)	0.941	0.542
Alcohol	141							
None		9(6.4)	5(11.1)	4(3.8)	4(8.2)	5(5.0)	0.173	0.476
Low		119(84.4)	37(82.2)	82(78.1)	41(83.7)	78(77.2)	0.947	0.252
High		13(9.2)	2(4.4)	11(10.5)	1(2.0)	12(11.9)	0.137	0.011
**Controls**	**N**	84(100)*	21(25)*	63(75)*	63(75)*	21(25)*		
Age (mean)	84	49.3	36.6	53.5	48.8	50.6	<0.001	0.453
Family CVD	84	49(58.3)	9(42.9)	40(63.5)	35(55.6)	14(66.7)	0.112	0.372
Diabetes	84	9(10.7)	1(4.8)	8(12.7)	5(7.9)	4(19.0)	0.218	0.249
Hypertension	84	16(19.0)	3(14.3)	13(20.6)	7(11.1)	9(42.9)	0.502	0.012
Dyslipidemia	84	12(14.3)	1(4.8)	11(17.5)	8(12.7)	4(19.0)	0.066	0.52
Smoking	84	57(67.9)	12(57.1)	45(71.4)	40(63.5)	17(81.0)	0.26	0.11
BMI > 25	80	45(56.3)	11(52.4)	34(54.0)	31(49.2)	14(66.7)	0.872	0.147
Alcohol	81							
None		4(4.9)	0(0.0)	4(6.3)	3(4.8)	1(4.8)	0.045	0.989
Low		74(91.4)	20(95.2)	54(85.7)	56(88.9)	18(85.7)	0.399	0.817
High		3(3.7)	1(4.8)	2(3.2)	2(3.2)	1(4.8)	0.79	0.757

Data presented as number (percentage) or mean. *Percentage of total population. y = years; CHD = Coronary heart disease; PAD = Peripheral artery disease; CVD = Cardiovascular disease; BMI = Body mass index.

### Risk factors

**Figure 1:**
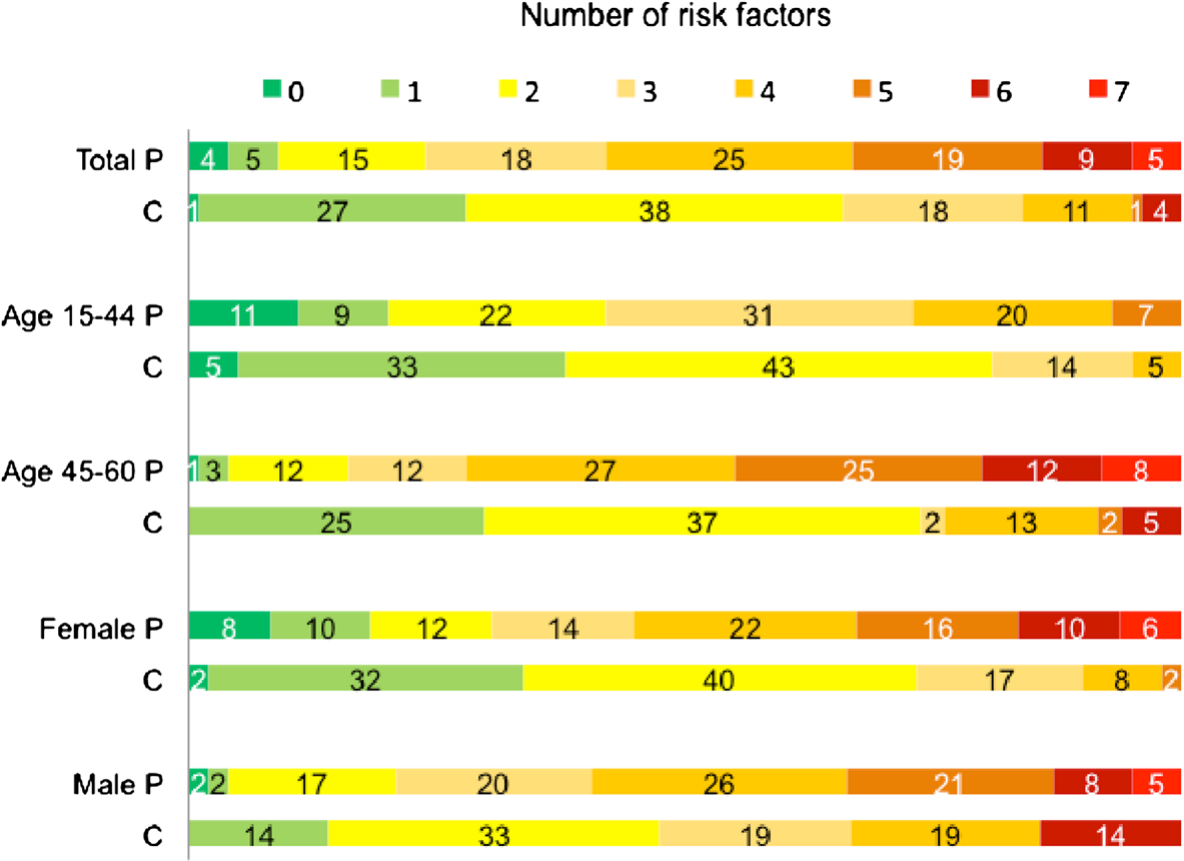
**Risk factor burden.** Data referred in percent. P = patients; C = controls.

Patients had a higher RF burden than controls in analysis of the entire study population (p < 0.001) and in subgroup analyses (age 15–44: p = 0.027; age 45–60: p < 0.001; females: p < 0.001; males: p = 0.021), visualized in Figure 1. Hypertension and dyslipidemia were more frequently present among patients. Prior stroke, CHD, family history of CVD, hypertension, dyslipidemia and smoking were less prevalent among young than among middle-aged patients, and smoking and high alcohol consumption were more common among male than among female patients.

### Carotid Intima-media thickness (cIMT)

Values of mean IMT and RC are presented in Table 2 and significance of all tests applied is shown in Table 3. Mean IMT values were in all subgroups of patients and controls lowest in CCA and highest in BIF, and lower in young than in middle-aged participants. Sex-related differences were inconsistent. Mean values <0.8 mm were mostly restricted to the young population and to controls. Mean values ≥1.0 mm were found in BIF in all patient and control subgroups but the young, and in ICA in middle-aged patients and male patients and controls. Mean values ≥1.5 mm were solely found in middle-aged patients.

**Table 2:** Relative change in IMT between patients and controls and mean IMT values

	**NA**	**Total**	**15-44 y**	**45-60 y**	**Females**	**Males**	**Age (p)**	**Sex (p)**
***CCA***								
Relative change		12 (4–22)	3 (−9-15)	19 (8–30)	4 (−6-15)	6 (−9-23)		
Mean IMT patients	1	0.85	0.63	0.94	0.75	0.89	<0.001	0.003
Mean IMT controls	0	0.73	0.61	0.77	0.7	0.82	<0.001	0.008
***BIF***								
Relative change		17 (2–34)	11 (−9-36)	23 (7–43)	9 (−9-31)	8 (−16-38)		
Mean IMT patients	11	1.34	0.84	1.54	1.23	1.39	<0.001	0.079
Mean IMT controls	2	1.12	0.7	1.26	1.04	1.34	<0.001	0.067
***ICA***								
Relative change		29 (12–49)	20 (−2-46)	35 (15–58)	28 (7–53)	1 (−23-31)		
Mean IMT patients	30	0.97	0.63	1.1	0.9	1.0	<0.001	0.312
Mean IMT controls	14	0.73	0.47	0.83	0.64	1.06	<0.001	0.021

Data sorted by carotid segment, age group, and sex. Relative change presented in % (CI); IMT presented in mm. CCA = Common carotid artery; BIF = Carotid bifurcation; ICA = Internal carotid artery; NA = Not available.

**Table 3:** Statistical significance for IMT increase in patients compared to controls

	**Total**	**15-44 y**	**45-60 y**	**Females**	**Males**
***CCA***					
Relative change (unadjusted)	**0.005**	0.669	**<0.001**	0.404	0.465
Mean IMT (t-test)	**<0.001**	0.501	**<0.001**	0.151	0.161
IMT distribution (Fisher’s exact test)	**0.018**	0.383	**0.003**	0.418	0.906
***BIF***					
Relative change (unadjusted)	**0.024**	0.299	**0.005**	0.344	0.544
Mean IMT (t-test)	**0.006**	**0.030**	**0.005**	0.108	0.760
IMT distribution (Fisher’s exact test)	0.111	0.447	**0.010**	0.202	**0.027**
***ICA***					
Relative change (unadjusted)	**<0.001**	0.079	**<0.001**	**0.008**	0.947
Mean IMT (t-test)	**<0.001**	**0.003**	**0.001**	**0.004**	0.741
IMT distribution (Fisher’s exact test)	**<0.001**	**0.005**	**<0.001**	**0.003**	0.155

Data presented as p-values, sorted by carotid segment, age group, and sex. Statistical significance presented in boldface. CCA = Common carotid artery; BIF = Carotid bifurcation; ICA = Internal carotid artery. Boldface represents statistical significant differences between patients and controls, mainly within the total population and among middle-aged participants, and as well among the young population and females in the ICA segment.

The RC between patients and controls was in all subgroups but males most distinct in ICA (20-35%). Only middle-aged patients showed IMT increase compared to controls in CCA (19%) and BIF (23%). Statistical simulation suggested the need of RC > 12% for CCA, RC > 22% for BIF, and RC > 25% for ICA to detect cIMT-differences ≥80% between patients and controls, which matches our results fairly well.

**Figure 2:**
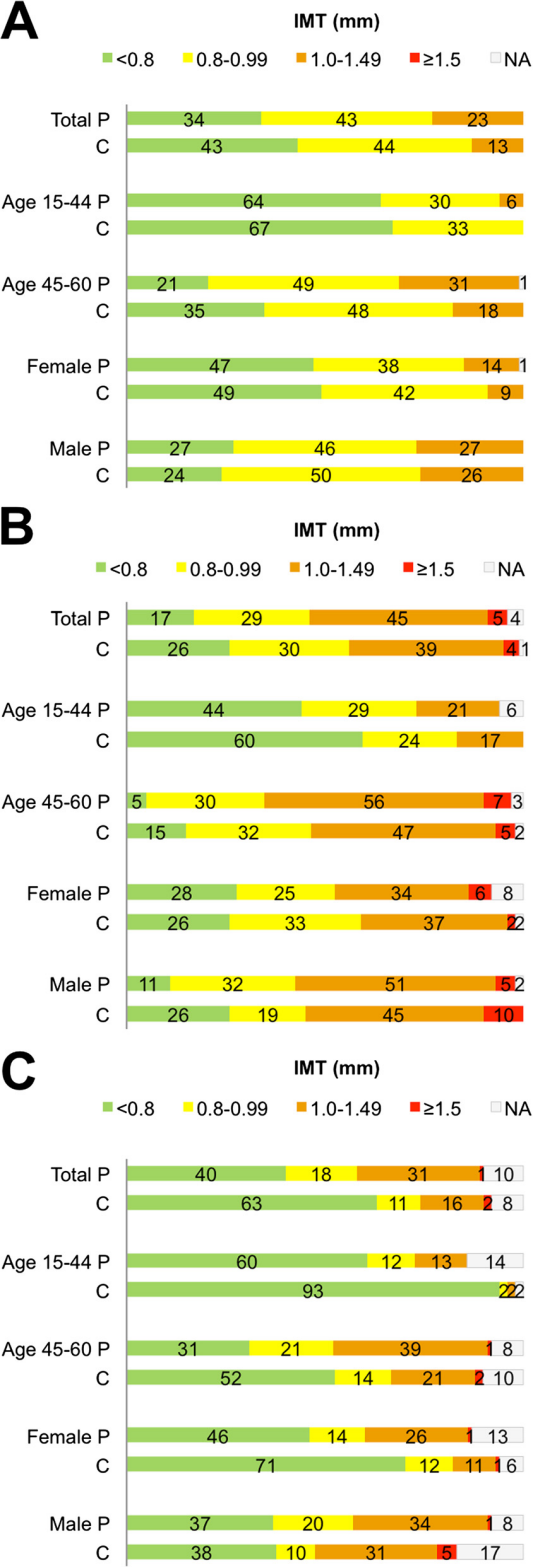
**Segmental cIMT distribution in common carotid artery, carotid bifurcation and Internal carotid artery.** Data referred in percent. **A** = Common carotid artery; **B** = Carotid bifurcation; **C** = Internal carotid artery; P = patients; C = controls.

Details on categorized segmental cIMT distribution are shown in Figure 2 and Table 3. IMT distribution was higher than that of controls in all patient subgroups but males in ICA. IMT distribution was further higher among middle-aged patients in CCA, and among middle-aged and male patients in BIF.

### Impact of risk factors on cIMT

Table 4 shows analysis of all participants (n = 234). Associations between RF and IMT increase varied in the different carotid segments, and across age and sex subgroups. With one exception (ICA in middle-aged participants), IMT increase was associated with age in all carotid segments among all subgroups. On the other hand, ICA in males was the only carotid segment in any subgroup, where patients’ RF had a stronger impact on IMT increase than controls’ (p = 0.038).

**Table 4:** Associations between vascular risk factors and segmental carotid IMT increase

**Participant group**	**Segment**	**Risk factor associations**
Entire study population (n = 234)	CCA:	Age (p < 0.001); male sex (p = 0.023); hypertension (p < 0.001)
	BIF:	Age (p < 0.001); dyslipidemia (p = 0.018); CHD (p = 0.017); smoking (p = 0.012)
	ICA:	Age (p < 0.001); dyslipidemia (p = 0.025); prior stroke (p = 0.009)
Age 15–44 years (n = 66)	CCA:	Age (p = 0.001); hypertension (p = 0.008)
	BIF:	Age (p < 0.001); hypertension (p = 0.009)
	ICA:	Age (p = 0.002); family history of CVD (p = 0.039)
Age 45–60 years (n = 168)	CCA:	Age (p = 0.05); male sex (p = 0.037); hypertension (p = 0.016)
	BIF:	Age (p = 0.022); dyslipidemia (p = 0.033); smoking (p = 0.016)
	ICA:	Dyslipidemia (p = 0.022); prior stroke (p = 0.037)
Females (n = 112)	CCA:	Age (p < 0.001); hypertension (p = 0.031)
	BIF:	Age (p < 0.001); hypertension (p = 0.049)
	ICA:	Age (p < 0.001)
Males (n = 122)	CCA:	Age (p < 0.001); hypertension (p = 0.012)
	BIF:	Age (p < 0.001); dyslipidemia (p = 0.003); CHD (p = 0.031)
	ICA:	Age (p < 0.001); dyslipidemia (p = 0.006), PAD (p = 0.028), prior stroke (p = 0.004)

CCA = Common carotid artery; BIF = Carotid bifurcation; ICA = Internal carotid artery.

## Discussion

Our study presents cIMT data obtained from young and middle-aged patients after acute ischemic stroke. Previous studies on multisegmental RF-cIMT associations related to incident stroke did either not include participants <45 years [21,22], or did not provide acute phase cIMT data [23]. By combination of three statistical methods, we identified increased ICA-IMT as distinctly associated with incident stroke not only among middle-aged adults, as others described before [21], but also among young adults aged 15–44 years. Patients aged 15–44 showed a surprising 20% ICA-IMT increase compared to controls. Only a family history of CVD was found to be associated with increased ICA-IMT in the younger subgroup, which may reflect a genetic predisposition [24].

Female patients represent the best-controlled subgroup in this study. We found an unexpected ICA-IMT increase of 28% compared to female controls. The increase was surprisingly high compared to a 4% increase in CCA-IMT, and a 9% increase in BIF-IMT. In comparison, we found rather low and constant segmental differences (1-8%) from controls in male patients, though their control group was less representative. The only factor related to ICA-IMT increase in females was age. Our results may reflect hormonal influences on the development of atherosclerosis [25-27]. ICA-IMT among male patients did not differ from controls’. However, male controls are insufficiently represented, what implies limitations for the interpretation of comparing results among males.

Our study shows a higher RF burden among patients, but also a high prevalence of RF among presumably healthy controls. Other studies have reported increasing risk of vascular events [28] and higher mortality proportional to RF burden [28-30], and recommended early preventive treatment. Our data strongly support broad preventive initiatives in families at risk.

We found an increasing RF burden with age, and the three most frequent RF were dyslipidemia (76%), smoking (69%) and hypertension (67%). This is in accordance with other studies [19,31]. Our RF rates among patients are, however, higher than previously reported [31], as e.g. cholesterol levels among the Norwegian population remain high despite improvement during the last decades [32]. Our data further support that cIMT depends on age, sex and cardiovascular risk [15,33-36]. We found pronounced cIMT increase in middle-aged patients, which is in line with a recent young stroke study demonstrating substantial clinical and subclinical atherosclerosis [20].

The major strengths of NOR-SYS are the inclusion of CVD-free controls and the standardized ultrasound protocol. However, our study has limitations. The size of patient subgroups varies as a consequence of stringent stratification of our patient population. Accordingly, controls (the patients’ partners) are unequally represented. Due to overall low case numbers, RF associations are in parts calculated with small sample sizes, which may have affected our results. Further, our data may be valid only for Caucasians, and may be influenced by the high risk profile of our population.

We did not account for multiple testing. However, as we performed approximately 100 tests, Bonferroni correction (BC) would yield a corrected significance level of about 0.05/100 = 0.0005, rounded to p < 0.001 in our study. Hence, associations with p < 0.001 would survive a BC. BC further reduces the number of type I errors at the cost of increasing the number of type II errors, and p-values above 0.0005 may still be indicative of an association.

## Conclusions

Stroke is associated with increased ICA-IMT already at a young age, related to a family history of CVD among the youngest patients and related to RF burden increasing with age. Also in CVD-free controls, RFs and subclinical atherosclerosis are prevalent. Our data suggest that vascular screening reveals established clinical and sub-clinical arterial disease requiring broad and aggressive treatment in order to prevent progressing CVD.

## Competing interests

The authors declare that they have no competing interests.

## Authors’ contributions

AF designed the study, carried out the ultrasound examinations and data aquisition, participated in statistical analysis and data interpretation, and drafted the manuscript. ØAH participated in the design of the study, performed statistical analysis and data interpretation and drafted the manuscript. HN contributed with acquisition of data and critical revision of the manuscript for intellectual content. LT contributed with acquisition of data and critical revision of the manuscript for intellectual content and helped to draft the manuscript. UWA contributed to study design, data aquisition, statistical analysis and data interpretation, critical revision of the manuscript for intellectual content and drafted the manuscript. All authors read and approved the final manuscript.

## References

[B1] Kappelle LJ Adams HP Jr Heffner ML Torner JC Gomez F Biller J Prognosis of young adults with ischemic stroke. A long-term follow-up study assessing recurrent vascular events and functional outcome in the Iowa Registry of Stroke in Young Adults Stroke 1994 25 7 1360 1365 8023350 10.1161/01.str.25.7.1360

[B2] Putaala J Haapaniemi E Metso AJ Metso TM Artto V Kaste M Tatlisumak T Recurrent ischemic events in young adults after first-ever ischemic stroke Ann Neurol 2010 68 5 661 671 21031581 10.1002/ana.22091

[B3] Waje-Andreassen U Naess H Thomassen L Eide GE Vedeler CA Arterial events after ischemic stroke at a young age: a cross-sectional long-term follow-up of patients and controls in western Norway Cerebrovasc Dis 2007 24 2–3 277 282 17646692 10.1159/000105680

[B4] Bots ML Grobbee DE Intima media thickness as a surrogate marker for generalised atherosclerosis Cardiovasc Drugs Ther 2002 16 4 341 351 12652104 10.1023/a:1021738111273

[B5] Iwakiri T Yano Y Sato Y Hatakeyama K Marutsuka K Fujimoto S Kitamura K Kario K Asada Y Usefulness of carotid intima-media thickness measurement as an indicator of generalized atherosclerosis: findings from autopsy analysis Atherosclerosis 2012 225 2 359 362 23092826 10.1016/j.atherosclerosis.2012.10.033

[B6] Lorenz MW Polak JF Kavousi M Mathiesen EB Volzke H Tuomainen TP Sander D Plichart M Catapano AL Robertson CM Kiechl S Rundek T Desvarieux M Lind L Schmid C DasMahapatra P Gao L Ziegelbauer K Bots ML Thompson SG PROG-IMT Study Group Carotid intima-media thickness progression to predict cardiovascular events in the general population (the PROG-IMT collaborative project): a meta-analysis of individual participant data Lancet 2012 379 9831 2053 2062 22541275 10.1016/S0140-6736(12)60441-3 PMC3918517

[B7] Den Ruijter HM Peters SA Anderson TJ Britton AR Dekker JM Eijkemans MJ Engstrom G Evans GW De Graaf J Grobbee DE Hedblad B Hofman A Holewijn S Ikeda A Kavousi M Kitagawa K Kitamura A Koffijberg H Lonn EM Lorenz MW Mathiesen EB Nijpels G Okazaki S O'Leary DH Polak JF Price JF Robertson C Rembold CM Rosvall M Rundek T Common carotid intima-media thickness measurements in cardiovascular risk prediction: a meta-analysis JAMA 2012 308 8 796 803 22910757 10.1001/jama.2012.9630

[B8] Fromm A Thomassen L Naess H Meijer R Eide GE Krakenes J Vedeler CA Gerdts E Larsen TH Kuiper KK Laxdal E Russell D Tatlisumak T Waje-Andreassen U The Norwegian Stroke in the Young Study (NOR-SYS): rationale and design BMC Neurol 2013 13 1 89 23865483 10.1186/1471-2377-13-89 PMC3721997

[B9] Bots ML Mulder PG Hofman A van Es GA Grobbee DE Reproducibility of carotid vessel wall thickness measurements. The Rotterdam Study J Clin Epidemiol 1994 47 8 921 930 7730896 10.1016/0895-4356(94)90196-1

[B10] Chambless LE Zhong MM Arnett D Folsom AR Riley WA Heiss G Variability in B-mode ultrasound measurements in the atherosclerosis risk in communities (ARIC) study Ultrasound Med Biol 1996 22 5 545 554 8865551 10.1016/0301-5629(96)00039-7

[B11] O’Leary DH Polak JF Kronmal RA Manolio TA Burke GL Wolfson SK Jr Carotid-artery intima and media thickness as a risk factor for myocardial infarction and stroke in older adults. Cardiovascular Health Study Collaborative Research Group N Engl J Med 1999 340 1 14 22 9878640 10.1056/NEJM199901073400103

[B12] Salonen R Haapanen A Salonen JT Measurement of intima-media thickness of common carotid arteries with high-resolution B-mode ultrasonography: inter- and intra-observer variability Ultrasound Med Biol 1991 17 3 225 230 1887507 10.1016/0301-5629(91)90043-v

[B13] Hollander M Hak AE Koudstaal PJ Bots ML Grobbee DE Hofman A Witteman JC Breteler MM Comparison between measures of atherosclerosis and risk of stroke: the Rotterdam Study Stroke 2003 34 10 2367 2372 12958327 10.1161/01.STR.0000091393.32060.0E

[B14] Aminbakhsh A Mancini GB Carotid intima-media thickness measurements: what defines an abnormality? A systematic review Clin Invest Med 1999 22 4 149 157 10497713

[B15] Chambless LE Heiss G Folsom AR Rosamond W Szklo M Sharrett AR Clegg LX Association of coronary heart disease incidence with carotid arterial wall thickness and major risk factors: the Atherosclerosis Risk in Communities (ARIC) Study, 1987–1993 Am J Epidemiol 1997 146 6 483 494 9290509 10.1093/oxfordjournals.aje.a009302

[B16] Najjar SS Scuteri A Lakatta EG Arterial aging: is it an immutable cardiovascular risk factor? Hypertension 2005 46 3 454 462 16103272 10.1161/01.HYP.0000177474.06749.98

[B17] Touboul PJ Hennerici MG Meairs S Adams H Amarenco P Bornstein N Csiba L Desvarieux M Ebrahim S Hernandez Hernandez R Jaff M Kownator S Naqvi T Prati P Rundek T Sitzer M Schminke U Tardif JC Taylor A Vicaut E Woo KS Mannheim carotid intima-media thickness and plaque consensus (2004-2006-2011). An update on behalf of the advisory board of the 3rd, 4th and 5th watching the risk symposia, at the 13th, 15th and 20th European Stroke Conferences, Mannheim, Germany, 2004, Brussels, Belgium, 2006, and Hamburg, Germany, 2011 Cerebrovasc Dis 2012 34 4 290 296 23128470 10.1159/000343145 PMC3760791

[B18] Putaala J Metso AJ Metso TM Konkola N Kraemer Y Haapaniemi E Kaste M Tatlisumak T Analysis of 1008 consecutive patients aged 15 to 49 with first-ever ischemic stroke: the Helsinki young stroke registry Stroke 2009 40 4 1195 1203 19246709 10.1161/STROKEAHA.108.529883

[B19] Von Sarnowski B Putaala J Grittner U Gaertner B Schminke U Curtze S Huber R Tanislav C Lichy C Demarin V Basic-Kes V Ringelstein EB Neumann-Haefelin T Enzinger C Fazekas F Rothwell PM Dichgans M Jungehulsing GJ Heuschmann PU Kaps M Norrving B Rolfs A Kessler C Tatlisumak T sifap1 Investigators Lifestyle risk factors for ischemic stroke and transient ischemic attack in young adults in the Stroke in Young Fabry Patients study Stroke 2013 44 1 119 125 23150649 10.1161/STROKEAHA.112.665190

[B20] von Sarnowski B Schminke U Tatlisumak T Putaala J Grittner U Kaps M Tobin WO Kinsella JA McCabe DJ Hennerici MG Fazekas F Norrving B Kessler C Rolfs A sifap1 investigators Prevalence of stenoses and occlusions of brain-supplying arteries in young stroke patients Neurology 2013 80 14 1287 1294 23468548 10.1212/WNL.0b013e31828ab2ed

[B21] Chambless LE Folsom AR Clegg LX Sharrett AR Shahar E Nieto FJ Rosamond WD Evans G Carotid wall thickness is predictive of incident clinical stroke: the Atherosclerosis Risk in Communities (ARIC) study Am J Epidemiol 2000 151 5 478 487 10707916 10.1093/oxfordjournals.aje.a010233

[B22] Hofman A Breteler MM van Duijn CM Janssen HL Krestin GP Kuipers EJ Stricker BH Tiemeier H Uitterlinden AG Vingerling JR Witteman JC The Rotterdam Study: 2010 objectives and design update Eur J Epidemiol 2009 24 9 553 572 19728115 10.1007/s10654-009-9386-z PMC2744826

[B23] Waje-Andreassen U Naess H Thomassen L Eide GE Meijer R Vedeler CA Ultrasound, atherosclerosis and stroke at a young age: a cross-sectional long-term follow-up in western Norway Eur J Neurol 2008 15 5 512 519 18355304 10.1111/j.1468-1331.2008.02118.x

[B24] Traylor M Farrall M Holliday EG Sudlow C Hopewell JC Cheng YC Fornage M Ikram MA Malik R Bevan S Thorsteinsdottir U Nalls MA Longstreth W Wiggins KL Yadav S Parati EA Destefano AL Worrall BB Kittner SJ Khan MS Reiner AP Helgadottir A Achterberg S Fernandez-Cadenas I Abboud S Schmidt R Walters M Chen WM Ringelstein EB O'Donnell M Genetic risk factors for ischaemic stroke and its subtypes (the METASTROKE collaboration): a meta-analysis of genome-wide association studies Lancet Neurol 2012 11 11 951 962 23041239 10.1016/S1474-4422(12)70234-X PMC3490334

[B25] Joakimsen O Bonaa KH Stensland-Bugge E Jacobsen BK Population-based study of age at menopause and ultrasound assessed carotid atherosclerosis: the Tromso Study J Clin Epidemiol 2000 53 5 525 530 10812326 10.1016/s0895-4356(99)00197-3

[B26] Witteman JC Grobbee DE Kok FJ Hofman A Valkenburg HA Increased risk of atherosclerosis in women after the menopause BMJ 1989 298 6674 642 644 2496790 10.1136/bmj.298.6674.642 PMC1835855

[B27] Wellons M Ouyang P Schreiner PJ Herrington DM Vaidya D Early menopause predicts future coronary heart disease and stroke: the Multi-Ethnic Study of Atherosclerosis Menopause 2012 19 10 1081 1087 22692332 10.1097/gme.0b013e3182517bd0 PMC3443540

[B28] Putaala J Haapaniemi E Kaste M Tatlisumak T How does number of risk factors affect prognosis in young patients with ischemic stroke? Stroke 2012 43 2 356 361 22052508 10.1161/STROKEAHA.111.635276

[B29] Gjerde G Naess H Risk factor burden predicts long-term mortality after cerebral infarction Acta Neurol Scand 2013 doi:10.1111/ane.12159. [Epub ahead of print] 10.1111/ane.12159 23803011

[B30] Naess H Waje-Andreassen U Nyland H Risk factor burden predicts long-term mortality in young patients with arterial cerebral infarction Acta Neurol Scand 2013 127 2 92 96 22616900 10.1111/j.1600-0404.2012.01681.x

[B31] Putaala J Yesilot N Waje-Andreassen U Pitkaniemi J Vassilopoulou S Nardi K Odier C Hofgart G Engelter S Burow A Mihalka L Kloss M Ferrari J Lemmens R Coban O Haapaniemi E Maaijwee N Rutten-Jacobs L Bersano A Cereda C Baron P Borellini L Valcarenghi C Thomassen L Grau AJ Palm F Urbanek C Tuncay R Durukan-Tolvanen A van Dijk EJ de Leeuw FE Demographic and geographic vascular risk factor differences in European young adults with ischemic stroke: the 15 cities young stroke study Stroke 2012 43 10 2624 2630 22798330 10.1161/STROKEAHA.112.662866

[B32] Jenum AK Graff-Iversen S Selmer R Selmer R Sogaard AJ Risk factors for cardiovascular disease and diabetes through three decades Tidsskr Nor Laegeforen 2007 127 19 2532 2536 17925822

[B33] De Groot E Hovingh GK Zwinderman AH Wiegman A Smit AJ Kastelein JJ Data density curves of B-mode ultrasound arterial wall thickness measurements in unaffected control and at-risk populations Int Angiol 2005 24 4 359 365 16355094

[B34] Polak JF Pencina MJ Meisner A Pencina KM Brown LS Wolf PA D’Agostino RB Sr Associations of carotid artery intima-media thickness (IMT) with risk factors and prevalent cardiovascular disease: comparison of mean common carotid artery IMT with maximum internal carotid artery IMT J Ultrasound Med 2010 29 12 1759 1768 21098848 10.7863/jum.2010.29.12.1759 PMC3186063

[B35] Bots ML Hoes AW Koudstaal PJ Hofman A Grobbee DE Common carotid intima-media thickness and risk of stroke and myocardial infarction: the Rotterdam Study Circulation 1997 96 5 1432 1437 9315528 10.1161/01.cir.96.5.1432

[B36] Howard G Manolio TA Burke GL Wolfson SK O’Leary DH Does the association of risk factors and atherosclerosis change with age? An analysis of the combined ARIC and CHS cohorts. The Atherosclerosis Risk in Communities (ARIC) and Cardiovascular Health Study (CHS) investigators Stroke 1997 28 9 1693 1701 9303011 10.1161/01.str.28.9.1693

